# Management of patient with acute lymphocytic myocarditis and congenital long QT syndrome presenting with electrical storm and incessant Torsade de Pointes: a case report

**DOI:** 10.1186/s13256-024-04919-z

**Published:** 2024-12-20

**Authors:** Giky Karwiky, Raymond Pranata, Alberta Claudia Undarsa, Mohamad Iqbal, Hawani Sasmaya Prameswari, Mohammad Rizki Akbar

**Affiliations:** Department of Cardiology and Vascular Medicine, Faculty of Medicine, Universitas Padjadjaran, Hasan Sadikin General Hospital, Bandung, Indonesia

**Keywords:** Myocarditis, Torsade de pointes, Long QT syndrome, Incessant ventricular tachycardia, Left cardiac sympathetic denervation

## Abstract

**Background:**

This case highlights the management of concomitant acute myocarditis and congenital long QT syndrome with electrical storm and incessant Torsade de Pointes.

**Case presentation:**

An 18 years-old Southeast Asian para 1 abortus 0 (P1A0) postpartum patient with cesarean section owing to severe preeclampsia, acute lymphocytic myocarditis, and prolonged QT interval owing to long QT syndrome. She has incessant Torsade de Pointes treated with beta-blocker, lidocaine, overdrive pacing with a temporary transvenous pacemaker, left cardiac sympathetic denervation per video-assisted thoracoscopic surgery, and implantable cardioverter-defibrillator implantation. We initially used bisoprolol, then switched to propranolol and finally to carvedilol, which reduced the Torsade de Pointes frequency. The longest QTc interval was 696 ms, and the shortest was 624 ms, 2 months after initial corticosteroid administration and left cardiac sympathetic denervation. Device interrogation at 9 months follow up showed three episodes of ventricular fibrillation, 2 spontaneously resolved and one necessitates shock.

**Conclusion:**

Management of concomitant acute myocarditis and congenital long QT syndrome with incessant Torsade de Pointes requires beta-blockers, anti-inflammatory drugs, autonomic modulation, and short-term measures, such as overdrive pacing with deep sedation. Implantable cardioverter-defibrillator is vital to prevent sudden cardiac death.

## Background

Prolonged QTc interval in patients with acute myocarditis is an independent predictor of fulminant course in acute myocarditis [1]. Inflammation and immune responses may trigger or exacerbate electrical instability in patients with congenital long QT syndrome (LQTS). Consequently, acute myocarditis may lead to incessant ventricular tachycardia (VT) or electrical storms [2]. Managing these patients often involves the use of anti-inflammatory medications and autonomic modulation.

This case highlights the management of concomitant acute myocarditis and congenital LQTS, complicated by electrical storms and incessant Torsade de Pointes (TdP), necessitating overdrive pacing, deep sedation, beta-blockers, corticosteroids, and eventually implantable cardioverter-defibrillator (ICD) implantation and left cardiac sympathetic denervation (LCSD).

## Case presentation

**Fig. 1:**
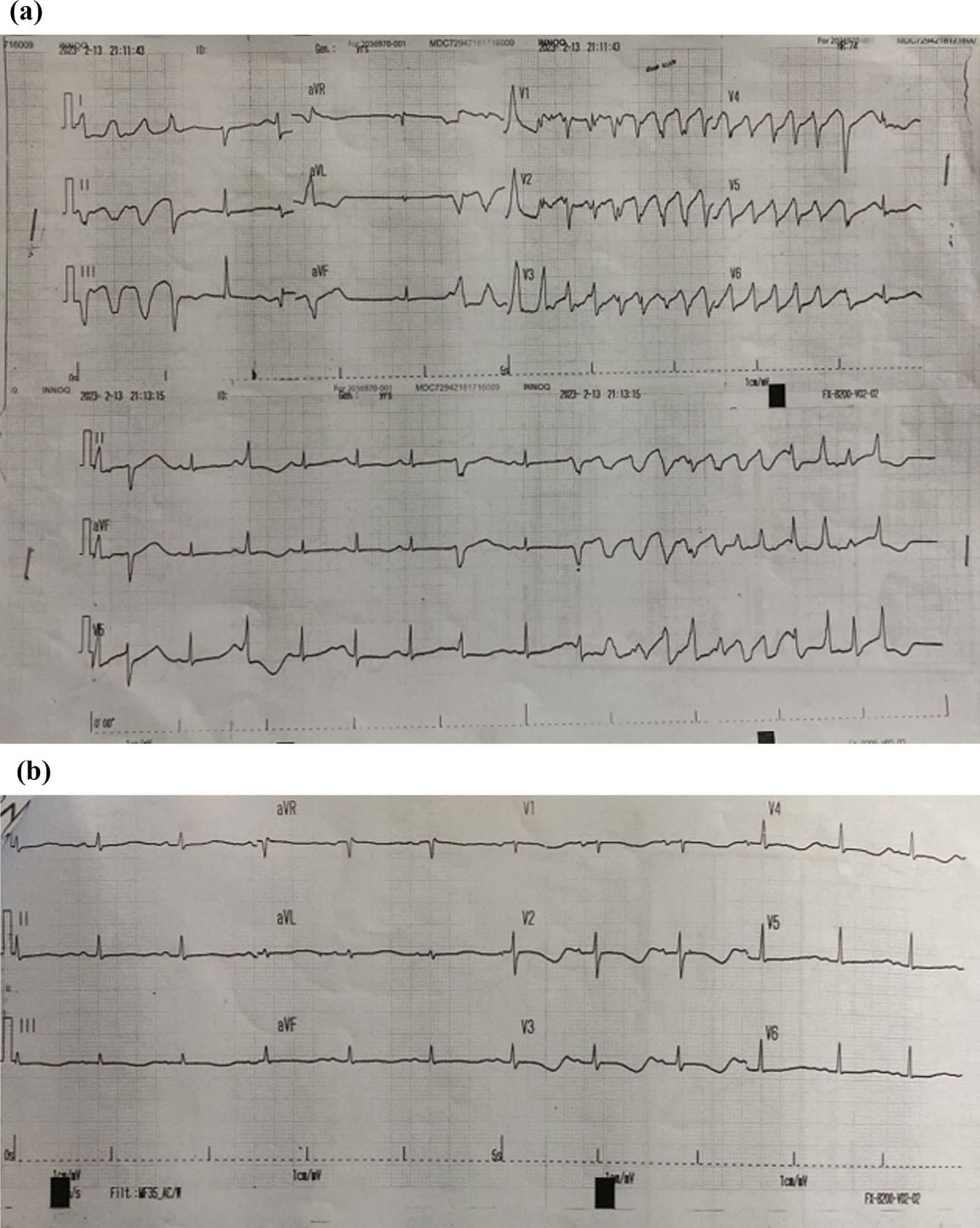
Electrocardiogram showing episodes of Torsade de Pointes (**A**) and sinus rhythm (post lidocaine) (**B**)

An 18-year-old Southeast Asian woman, para 1 abortus 0 (P1A0), presented with incessant VT and several pulseless episodes. She was 1 week postpartum following a cesarean section owing to severe preeclampsia. At 2 weeks before admission, she experienced shortness of breath. Laboratory tests revealed anemia with normal renal function and liver enzymes (alanine aminotransferase and aspartate aminotransferase). A chest X-ray indicated cardiomegaly. A 12-lead electrocardiogram (ECG) displayed episodes of TdP (Fig. 1A), and during sinus rhythm, the patient had a prolonged QTc interval with a maximum of 696 ms (Fig. 1B). Despite treatment with magnesium sulfate (MgSO4) and lidocaine (1 mg/min), persistent VT required sedation, intubation, and overdrive pacing with a temporary transvenous pacemaker. Serum electrolytes, thyroid stimulating hormone, T3, and T4 levels were within normal limits.

**Fig. 2:**
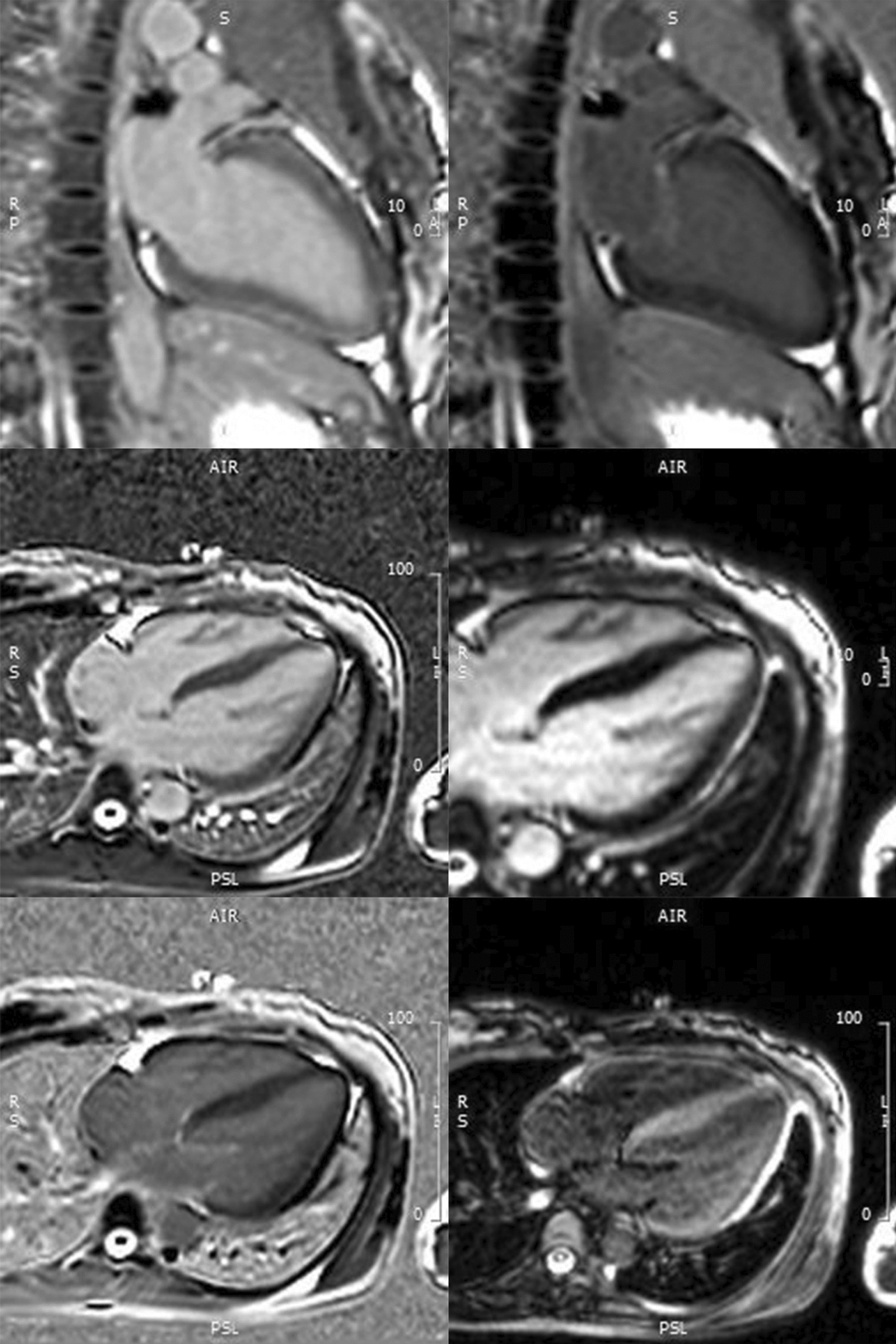
Cardiac magnetic resonance imaging showing subepicardial fibrosis at basal-mid anterior, mid lateral, basal inferior; intramyocardial fibrosis at mid septal; patchy fibrosis at mid posterior right-left ventricular junction and apico-inferoseptal wall consistent with myocarditis

Echocardiography showed a dilated left atrium and left ventricle (LV), eccentric LV hypertrophy, reduced LV systolic function (LVEF 46%) with global hypokinesis, grade II LV diastolic dysfunction, mild functional mitral regurgitation, and normal valve anatomy. The right ventricular systolic function was normal, with intermediate pulmonary hypertension (PH) probability. Cardiac magnetic resonance imaging (MRI) showed subepicardial fibrosis in the basal-mid anterior, mid-lateral, and basal inferior regions; intramyocardial fibrosis in the mid-septal region; patchy fibrosis in the mid-posterior right-left ventricular (LV) junction and apico-inferoseptal wall; and increased T2 intensity consistent with myocarditis (Fig. 2). Bilateral hilar lymphadenopathy raised the possibility of sarcoidosis, but an endomyocardial biopsy confirmed lymphocytic myocarditis. The patient was diagnosed with lymphocytic myocarditis, prolonged QT interval owing to congenital LQTS, and P1A0 postpartum status following a cesarean section owing to severe preeclampsia. A summary of diagnostic findings is presented in Table 1.

**Table 1:** Summary of diagnostic findings

Diagnostic tests	Findings
EKG	Prolonged QTc interval (longest 696 ms)
Echocardiography	Dilated LA, LV, eccentric LVH Reduced LV systolic function (LVEF 46%) with global hypokinetic LV diastolic dysfunction grade II Mild functional mitral regurgitation, normal anatomy, and function of other valves Intermediate probability of PH Normal RV systolic function
Cardiac MRI	Subepicardial fibrosis at basal-mid anterior, mid lateral, basal inferior Intramyocardial fibrosis at mid septal Patchy fibrosis at mid posterior RV-LV junction and apico-inferoseptal wall along with increased T2 intensity Findings were consistent with myocarditis
Endomyocardial biopsy	Lymphocytic infiltrate consistent with lymphocytic myocarditis

PH: pulmonary hypertension, MRI: magnetic resonance imaging, RV: right ventricle, LA: left atrium, LV: left ventricle, LVEF: left ventricular ejection fraction, LVH: left ventricular hypertrophy

**Fig. 3:**
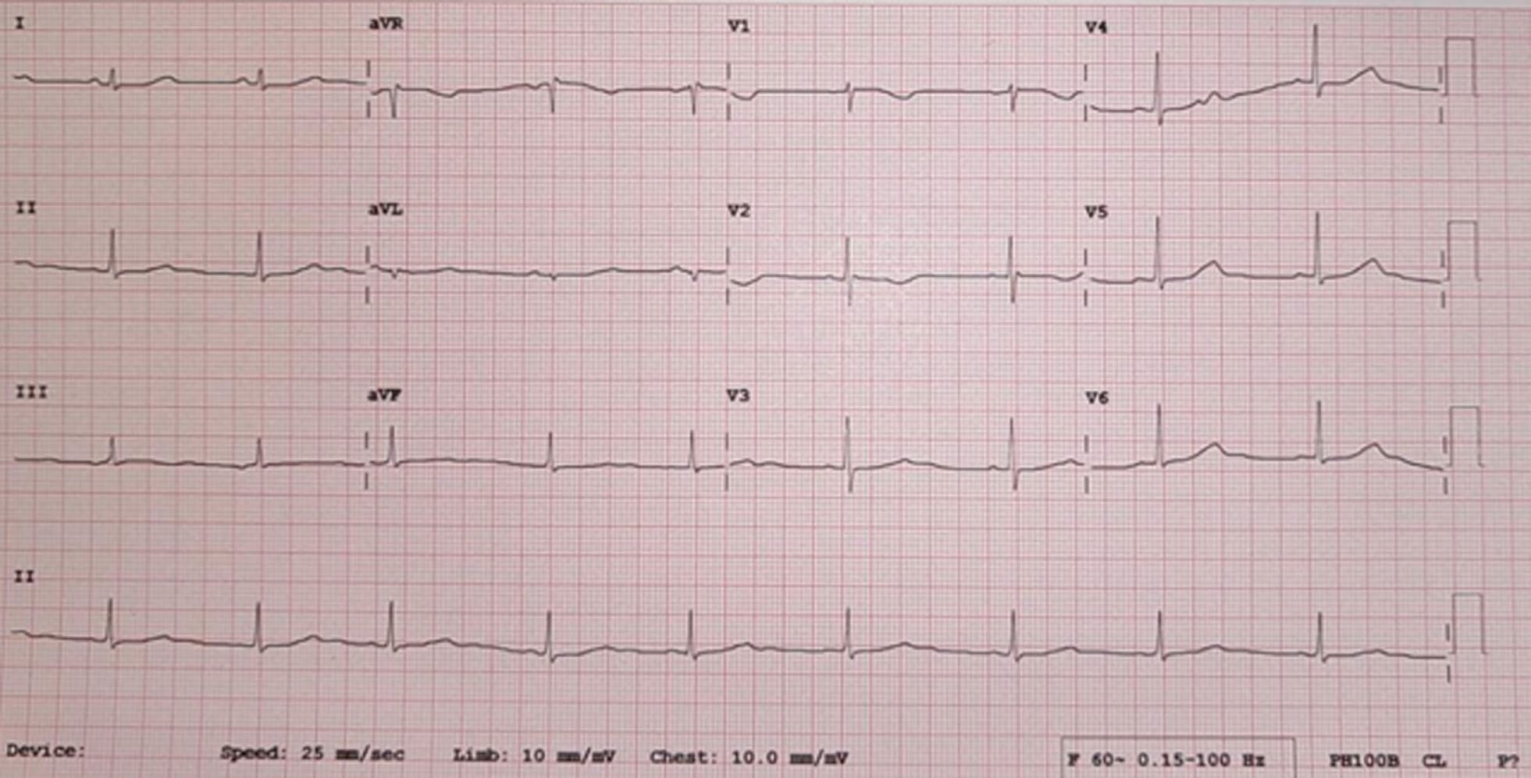
Electrocardiogram on 2 months follow-up

The patient was treated with prednisone (40 mg/day), lisinopril (2.5 mg/day), heparin (5000 units twice daily), and lansoprazole (30 mg intravenously daily). Initially, bisoprolol (10 mg/day) was prescribed, but persistent VT led to a switch to propranolol (40 mg three times daily), the preferred treatment for electrical storms. However, owing to the high burden of premature ventricular contractions (PVCs) and VT, propranolol was replaced with carvedilol (25 mg twice daily) on the basis of studies suggesting carvedilol’s superiority in preventing cardiac events compared with other beta-blockers. The patient underwent LCSD via video-assisted thoracoscopic surgery and ICD implantation. Following LCSD, the patient experienced no further episodes of PVC or VT, and the QTc interval reduced to 583 ms. At a 1.5-month follow-up, an ECG showed sinus bradycardia with a QTc of 624 ms (Fig. 3). Device interrogation at a 9-month follow-up revealed three episodes of ventricular fibrillation (VF), two of which resolved spontaneously, while one required shock therapy. The patient reported no symptoms outside of the recorded events, indicating no missed VF detection.

## Discussion and conclusion

This case describes an 18-year-old postpartum woman with a history of severe preeclampsia who developed acute lymphocytic myocarditis and a prolonged QT interval owing to congenital LQTS. She experienced electrical storms and incessant TdP despite lidocaine and sedation, leading to the use of overdrive pacing using a temporary transvenous pacemaker. LCSD was performed to reduce the arrhythmic burden, and an ICD was implanted for secondary prevention.

The patient was referred to us with a suspicion of peripartum cardiomyopathy. Echocardiography showed reduced LV systolic function with global hypokinetic. However, VT rarely occurs in peripartum cardiomyopathy (4.2%) compared with acute myocarditis (approximately 8.3%) [3, 4], thus we perform work-up to establish/rule-out myocarditis.

In this case, myocarditis may have triggered TdP in a patient with congenital LQTS. Additionally, polymorphic PVCs and VT are more common in the active inflammatory phase of myocarditis and reflect the dynamic nature of arrhythmogenic substrate, suggesting multifocal origin [5, 6]. The PVCs likely triggered TdP in the context of the prolonged QT interval, possibly driven by direct myocardial injury and increased sympathetic activity in response to acute heart failure. Systemic inflammation may increase susceptibility to arrhythmia owing to circulating cytokines affecting cardiomyocyte action potential duration and increasing sympathetic output from the central and peripheral autonomic nervous system [2]. Myocarditis itself may cause prolonged QTc, it may occur in up to 25% of the cases and is a predictor of death [2, 7]. Patients with fulminant myocarditis during treatment shortened QRS duration and QTc interval among survivors [1].

Inflammation and immunity may cause clinical expression of congenital LQTS, which may trigger or enhance electrical instability in genetically predisposed patients [2]. Study by Rizzo *et al*. that performed LCSD in patients with intractable ventricular arrhythmias showed that stellate ganglion specimens showed T cell and macrophage-mediated inflammations, which may increase adrenergic activity or enhance electrical instability in patients with a genetic predisposition to arrhythmia [8]. Thus, we performed cardiovascular magnetic resonance (CMR) and endomyocardial biopsy to obtain a diagnosis and determine whether corticosteroid is needed. Biopsy showed lymphocytic myocarditis, and we give high dose corticosteroid.

Our patient experienced VT despite initial bisoprolol treatment, leading to a switch to propranolol. Owing to ongoing VT recurrence, the medication was further changed to carvedilol. Not all beta-blockers are equal in patients with LQTS, a study showed that propranolol has a more significant QTc-shortening effect and breakthrough cardiac event prevention than metoprolol and nadolol [9]. Metoprolol is not recommended in LQTS type 2, since this patient has double peak T wave (T and U wave) we did not use metoprolol in this patient [9]. Kimura *et al*. compared selective beta blockers (51% was propranolol) and carvedilol in patients with LQTS type 2, cardiac event was found in 26% in former and 0% in the latter (*p* = 0.098) [10]. Additional α1-adrenoceptor blockade in carvedilol may suppress QT prolongation and/or transmural dispersion of repolarization and TdP [10]. VT recurrence was significantly reduced in our patient after we switched to carvedilol.

Deep sedation was administered to reduce sympathetic activity, and 80% of the patients achieved complete resolution of VT/VF within minutes to hours [11]. Overdrive pacing was used to avoid defibrillation owing to TdP and has a class I indication (ESC Guideline) in LQTS with electrical storm [12]. LCSD was performed to protect from arrhythmia by complete sympathetic blockade [13, 14], thus reducing VT recurrence and ICD shocks in patients with LQTS. LCSD may also reduce QTc interval by an average of 60 ms in 50% of patients with LQTS and QTc ≥ 500 ms [14]. Dusi *et al*. recommend LCSD in patients with high risk of recurrences despite beta-blocker use and aborted cardiac arrest patients treated with beta-blockers and have QTc ≥ 500 ms [14]. Autonomic modulation has a Class IIa indication (ESC guideline) in the management of electrical storm owing to polymorphic ventricular arrhythmia [12]. Additionally, a preclinical study showed LCSD attenuates myocardial inflammation, albeit in post-myocardial infarction mice [15].

ICD implantation in this patient was based on cardiac arrest and persistent TdP despite beta-blocker during hospitalization. Hemodynamically not-tolerated VT/VF during the acute phase of myocarditis met class IIa recommendation (ESC 2022) for ICD implantation and cardiac arrest in LQTS met Class I recommendation (ESC 2022, AHA/ACC/HRS 2017) on the basis of the guidelines [12].

Prolonged QT interval does not resolve after 2 months of corticosteroid regimen and use of LCSD, although the QTc interval is shortened to 624 ms. Additionally, the longest QTc interval was 696 ms; thus, the prolonged QT is likely owing to congenital LQTS and the episodes of arrhythmia triggered by acute myocarditis.

In conclusion, managing concomitant acute myocarditis and congenital LQTS with electrical storms and incessant TdP requires a combination of overdrive pacing, deep sedation, beta-blockers, and corticosteroids. ICD implantation is essential for preventing sudden cardiac death, and LCSD plays a key role in reducing recurrent TdP and ICD shocks. Regular ICD interrogation is crucial for optimizing treatment, and substrate ablation may be required if appropriate shocks recur despite optimal medical therapy.

## Data Availability

Available upon reasonable request to the corresponding author.

## Abbreviations

CSD: Cardiac sympathetic denervation
ICD: Implantable cardioverter-defibrillator
LCSD: Left cardiac sympathetic denervation
LQTS: Long QT syndrome
LV: Left ventricle
PVC: Premature ventricular complex
RV: Right ventricle
TdP: Torsade de Pointe
VF: Ventricular fibrillation
VT: Ventricular tachycardia
